# Skull shape of a widely distributed, endangered marsupial reveals little evidence of local adaptation between fragmented populations

**DOI:** 10.1002/ece3.6593

**Published:** 2020-08-18

**Authors:** Pietro Viacava, Simone P. Blomberg, Gabriele Sansalone, Matthew J. Phillips, Thomas Guillerme, Skye F. Cameron, Robbie S. Wilson, Vera Weisbecker

**Affiliations:** ^1^ School of Biological Sciences The University of Queensland St. Lucia QLD Australia; ^2^ Form, Evolution and Anatomy Research Laboratory, Zoology School of Environmental and Rural Sciences University of New England Armidale NSW Australia; ^3^ Earth, Environmental and Biological Sciences School Queensland University of Technology Brisbane QLD Australia; ^4^ College of Science and Engineering Flinders University Adelaide SA Australia

**Keywords:** conservation, *Dasyurus hallucatus*, geometric morphometrics, intraspecific variation, procrustes ANOVA, shape variation, variation partitioning

## Abstract

The biogeographic distribution of diversity among populations of threatened mammalian species is generally investigated using population genetics. However, intraspecific phenotypic diversity is rarely assessed beyond taxonomy‐focused linear measurements or qualitative descriptions. Here, we use a technique widely used in the evolutionary sciences—geometric morphometrics—to characterize shape diversity in the skull of an endangered marsupial, the northern quoll, across its 5,000 km distribution range along Northern Australia. Skull shape is a proxy for feeding, behavior, and phenotypic differentiation, allowing us to ask whether populations can be distinguished and whether patterns of variation indicate adaptability to changing environmental conditions. We analyzed skull shape in 101 individuals across four mainland populations and several islands. We assessed the contribution of population, size, sex, rainfall, temperature, and geography to skull shape variation using principal component analysis, Procrustes ANOVA, and variation partitioning analyses. The populations harbor similar amounts of broadly overlapping skull shape variation, with relatively low geographic effects. Size predicted skull shape best, coinciding with braincase size variation and differences in zygomatic arches. Size‐adjusted differences in populations explained less variation with far smaller effect sizes, relating to changes in the insertion areas of masticatory muscles, as well as the upper muzzle and incisor region. Climatic and geographic variables contributed little. Strikingly, the vast majority of shape variation—76%—remained unexplained. Our results suggest a uniform intraspecific scope for shape variation, possibly due to allometric constraints or phenotypic plasticity beyond the relatively strong allometric effect. The lack of local adaptation indicates that cross‐breeding between populations will not reduce local morphological skull (and probably general musculoskeletal) adaptation because none exists. However, the potential for heritable morphological variation (e.g., specialization to local diets) seems exceedingly limited. We conclude that 3D geometric morphometrics can provide a comprehensive, statistically rigorous phenomic contribution to genetic‐based conservation studies.

## Graphical abstract

The diversity among populations of threatened mammalian species is generally investigated through population genetics. Here, we characterize shape intraspecific diversity with geometric morphometrics in the skull of an endangered marsupial. Our results suggest a uniform within‐species scope for shape variation, possibly due to phenotypic plasticity or allometric constraints. Thus, the potential for heritable morphological variation, such as specialization to local diets, seems exceedingly limited.

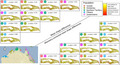

## 1 INTRODUCTION

The conservation of mammalian diversity is an urgent global issue (Bowyer, Boyce, Goheen, & Rachlow, 2019; Crooks et al., 2017), but population declines have been particularly precipitous in Australian marsupials (Baker & Dickman, 2018; Fisher et al., 2014; Woinarski, Burbidge, & Harrison, 2015; Ziembicki et al., 2015). A high proportion of marsupials reached high levels of vulnerability in the last century, making them a particular conservation concern (Woinarski et al., 2011). Consequently, high priority conservation efforts are underway for over one hundred threatened Australian mammals (Legge et al., 2018).

One of the challenges of current conservation efforts is the determination of within‐species diversity. Determining population units for management plans ensures the preservation of evolutionary potential in endangered species (Crandall, Bininda‐Emonds, Mace, & Wayne, 2000; Moritz, 1994) and the functioning of ecosystems (Des Roches et al., 2018). Population units are largely determined using molecular data (Allendorf, 2017)—for example, researchers of endangered species of squirrels (Finnegan, Edwards, & Rochford, 2008), jaguars (Wultsch et al., 2016), and wolves (Hindrikson et al., 2017) have all relied on genetics to identify their population diversity for conservation purposes. This genetic management has established links between diversity metrics and population fitness; however, it does not assess the phenotypic variation within a species and therefore does not discriminate this aspect of the organismic diversity within a population (Wanninger, 2015). This results in the potential for serious disjuncts between phenotypic intraspecific variation and genotype variability (Boyko et al., 2010; Le Rouzic & Carlborg, 2008; Vogt et al., 2008).

Understanding the phenotypic diversity of fragmented populations can provide valuable information to conservation management. In particular—in analogy to the interpretation of genetic distances—morphological differences between populations may indicate local adaptation (Colangelo et al., 2012; Meloro, 2011; Meloro, Guidarelli, Colangelo, Ciucci, & Loy, 2017). Current conservation studies of endangered taxa rarely use morphological data to determine phenotypic differentiation below the species level (Dierickx, Shultz, Sato, Hiraoka, & Edwards, 2015; Wilting et al., 2015); and most quantitatively rigorous assessment of phenotypic differentiation remains the domain of taxonomic studies (Celik et al., 2019; Meloro et al., 2017; Nicolosi & Loy, 2019; Senczuk et al., 2018; Sveegaard et al., 2015). Therefore, quantifying morphological variation within a species represents a largely untapped potential for understanding the phenotypic variation between taxonomic units and testing of hypotheses of adaptation and relatedness within a species. In addition, the morphological diagnosis of populations provides a valuable tool for management, for example, in assessing whether population units may be too morphologically divergent to be crossbred in outbreeding conservation efforts. It can also inform predictions of morphological change during future species’ fragmentation events, which is a common consequence of human activity (Bennett & Saunders, 2010; Haddad et al., 2015; Hansen et al., 2013).

The anatomical complex with the most comprehensive amount of quantifiable morphological information is the mammalian skull. This is reflected in a long tradition of using linear skull measurements for taxonomic purposes (Baker, Mutton, Mason, & Gray, 2015; A. Cardini, 2013; Travouillon, 2016; Van Dyck, 2002). The shape of mammalian skulls contains information on animal function (Hanken & Hall, 1993), such as masticatory loading (Herring, Rafferty, Liu, & Marshall, 2001), acting as a proxy for dietary preferences in mammals (Maestri, Patterson, Fornel, Monteiro, & de Freitas, 2016; Marroig & Cheverud, 2005; Nogueira, Peracchi, & Monteiro, 2009), including marsupials (Mitchell, Sherratt, Ledogar, Sherratt, Ledogar, & Wroe, 2018; Wroe & Milne, 2007). This is particularly relevant in the context of marsupial mammals, whose skull might not be as adaptable as that of placental mammals due to a developmental constraint on skull shape variation (Goswami, Polly, Mock, & Sanchez‐Villagra, 2012; Porto, Shirai, de Oliveira, & Marroig, 2013; Sánchez‐Villagra, Goswami, Weisbecker, Mock, & Kuratani, 2008; Weisbecker, Goswami, Wroe, & Sanchez‐Villagra, 2008; Weisbecker et al., 2019). This is because marsupials are born at an extremely immature (altricial) state of development, but with a highly developed oral apparatus adapted to immediate and extensive feeding at the mother's teat. This seems to reduce the potential of the oral region to diversify, both developmentally (Goswami et al., 2016) and evolutionarily (Porto et al., 2013; Sánchez‐Villagra et al., 2008; Weisbecker et al., 2008). Such a developmental constraint may reduce the ability of the marsupial skull to adapt at the level of within‐species variation, leaving adaptation through changes in size as the only source of heritable adaptive shape variation (Marroig & Cheverud, 2005, 2010; Porto et al., 2013; Shirai & Marroig, 2010).

In this study, we use geometric morphometric analyses to provide the first population‐level study of variation in the morphology of the skull of a mammal of particular conservation concern. We focus on the endangered northern quoll (*Dasyurus hallucatus*: Gould, 1842), a small carnivorous marsupial (usually weighing between 300 and 900 g) (Oakwood, 1997) with well‐understood genetic differentiation among populations (Cardoso et al., 2009; Firestone, Houlden, Sherwin, & Geffen, 2000; Hill & Ward, 2010; Hohnen et al., 2016; How, Spencer, & Schmitt, 2009; Woolley, Krajewski, & Westerman, 2015) but no information on morphological adaptation of the skull. Northern quolls appear to have had a precolonial distribution over 5,000 km across northern Australia (Braithwaite & Griffiths, 1994). They are now separated by major biogeographic breaks into four mainland populations with no apparent gene flow (Hill & Ward, 2010) and several island populations (Woinarski et al., 1999). Northern quolls are also a suitable study system for this investigation because they inhabit a wide range of habitats, ranging from rainforests to deserts (Begg, 1981; Moore et al., 2019; Oakwood, 2002). They are opportunistic foragers of small vertebrates, invertebrates, fruit, and carrion (Dunlop, Rayner, & Doherty, 2017). The species is also expected to evolve quickly because, as a semelparous species, most males die off in their first year after mating (Oakwood, Bradley, & Cockburn, 2001).

We capture fine‐scale morphological differences of the cranium using 3D geometric morphometrics, which differs from traditional taxonomic morphometrics (Baker & Van Dyck, 2015; Travouillon et al., 2019) by being agnostic to expected shape differences and by allowing the size and shape variation of the whole skull to be described in high detail (Chaplin, Sumner, Hipsley, & Melville, 2020; Galatius, Kinze, & Teilmann, 2012; Milenvić, Šipetić, Blagojević, Tatović, & Vujošević, 2010; Sztencel‐Jabłonka, Jones, & Bogdanowicz, 2009). This process also has the ability to provide statistical analyses of shape variation patterns alongside visualizations of the major axes of shape variation, thus permitting a finely resolved examination of the drivers of shape variation that is not possible with conventional linear measurements.

We examine several potential factors that might impact on northern quoll skull shape variation. Given the discrete distribution of populations across Northern Australia, we expect shape differences between populations to be a main part of overall skull variation. If this variation relates to heritable adaptation to local environments, for example, through dietary differences between high and low rainfall areas (Dunlop et al., 2017), we expect differences between populations to increase with local environmental differences (such as rainfall and temperature). Alternatively, if local adaptation is limited by a constraint due to the quoll's early timing of birth, most shape variation is expected along a size gradient, rather than a population or climatic gradient, or might be unexplained. Such a result would suggest a one‐to‐many mapping scenario, possibly under stabilizing selection, where similarly sized individuals can fulfill multiple functions specific to their environment with one shape. It is also possible that most variation relates to the biomechanical use of the cranium due to their generalist feeding habits; this would be apparent from a strong first main axis of variation (principal component 1) which highlights areas involved in mastication—such as the zygomatic arch or incisor region—as varying most.

## 2 MATERIALS AND METHODS

### 2.1 Data collection (3D acquisition)

We reconstructed in silico 101 crania of adult individuals of *Dasyurus hallucatus*—including males and females from four mainland populations: Queensland (*n* = 35), Northern Territory (*n* = 31), Pilbara (*n* = 15), and Kimberley (*n* = 8); and island populations: Groote Eylandt (*n* = 7) and other small islands (*n* = 5). Adult status was determined through incisor wear (Oakwood, 2000) and P3 eruption (Woolley, Haslem, & Westerman, 2013). We 3D‐scanned most of the specimens from museum collections (Queensland Museum, Australian Museum, Western Australian Museum, Australian National Wildlife Collection and American Museum of Natural History) with a GoMeasure 3D HDI109 blue light surface scanner (LMI Technologies Inc., Vancouver, Canada). Each cranium was placed in 3 different orientations on a motorized rotary table that turned every 45 degrees (8 rotations per round). The 24 resulting 3D images (8 rotations x 3 orientations) were then meshed together with the scanner's software (flexscan3d 3.3) to export a complete 3D image of each skull. This file was then treated for postprocessing cleaning (so as to not affect the biological shape of the structure), mesh decimation (to facilitate computation), and mesh reformatting (as “.ply” files need to be in binary format for subsequent importations of the mesh into R). Several photographs of each specimen were also taken to help identify landmarks by distinguishing biological structures from 3D artefacts in the landmarking process. Seven fully fleshed specimens from the Groote Eylandt population were CT‐scanned at the Centre for Advanced Imaging at The University of Queensland in a micro‐CT scanner. In order to obtain the 3D model, segmentation of the DICOM grayscale images provided by the micro‐CT scan was performed with mimics research version 20.0. All 3D models can be accessed through MorphoSource. The University of Queensland animal ethics committee (permit number SBS/009/16/ARC) and the Northern Territory Parks and Wildlife Commission (permit number 58566) approved the research methods and the collection of the Groote Eylandt specimens.

### 2.2 Template creation

The template consists of 900 landmarks: 101 fixed landmarks, 93 curves (271 semilandmarks), and 18 surfaces (528 semilandmarks; Figure S1 and Table S1). The number of semilandmarks on curves or surfaces was determined by the complexity (inflection points) of the curves or area covered. High‐density landmark and semilandmark configurations, ranging from 800 to more than 1,000 landmarks, have been demonstrated empirically to successfully capture genuine biological signal (Cornette, Baylac, Souter, & Herrel, 2013; Dumont et al., 2016; Goswami et al., 2019; Gunz & Mitteroecker, 2013; Segall, Cornette, Fabre, Godoy‐Diana, & Herrel, 2016; Watanabe et al., 2019; Weisbecker et al., 2019).

To ensure the repeatability of landmarking of the manually placed fixed landmarks, three morphologically close specimens were digitized ten times. Measurement error was much lower than interindividual variation, confirming the high repeatability of the template used in this study (Figure S2).

### 2.3 Landmarking and sliding

Each 3D model was landmarked in viewbox version 4.0 (dHAL software, Kifissia, Greece; http://www.dhal.com; Polychronis *et al*., 2013). One operator (first author) manually placed the fixed landmarks and curves. Curve semilandmarks were placed equidistantly and then were allowesd to slide along their respective curves. Surface semilandmarks were placed following a thin‐plate spline interpolation between the template and each specimen, followed by a projection to the surface of the 3D model and the sliding procedure. Sliding was performed by minimizing bending energy.

### 2.4 Analysis

Raw coordinate data were analyzed in R version 3.6.1 (R Core Team, 2019) with the “geomorph” (version 3.1.2) (Adams & Otárola‐Castillo, 2013) and the “Morpho" (version 2.7) (Schlager, Jefferis, Ian, & Schlager, 2019) packages. A generalized Procrustes analysis (GPA) was performed on the raw landmarks to translate, rotate, and scale specimens to the same size. This allowed us to extract the size component as centroid size (Rohlf & Slice, 1990) and to analyze *shape* (form minus size) (Kendall, 1989). This GPA step was used for all specimens as well as subsets (e.g., if only specimens of known sex or mainland‐only specimens were considered for corresponding analyses). Despite its small sample size, we included specimens from Groote Eylandt (*n* = 7) as a separate population for all our analyses, taking into consideration this caveat in our interpretation of the results. We did not include the specimens from four other island populations for population analyses (total *n* = 5). We did, however, test whether there were differences in shape variation among all island (including Groote) and mainland individuals, which would occur if divergent selection on the different islands shaped each population differently.

#### 2.4.1 Morphological differences between populations

In order to explore the variation of shape in our dataset, we conducted a principal component analysis (PCA) on the landmark coordinates. This method reduces the large dimensionality of the dataset—due to the large number of variables (i.e., landmark coordinates)—by tracing orthogonal axes along the main variance–covariance axis of the data, with the result being that the first axes (i.e., principal components) represent most of the shape variation. If the identity of a population determines shape variation in the sample, one of the main principal components (PCs) is therefore expected to separate specimens according to populations.

We also assessed for shape differences between populations with Procrustes ANOVAs using the permutational procedure (1,000 iterations) implemented in the “geomorph” R package (Adams & Otárola‐Castillo, 2013) and then performed permutation‐based (1,000 iterations) pairwise comparisons between the shape, size‐corrected shape, and centroid size least squares means of each population (Collyer, Sekora, & Adams, 2015). We adjusted p‐values of pairwise comparisons with the Bonferroni method. Heat plot visualizations of mean comparisons between populations were used to understand where the main shape differences were located. We executed all heat plot visualizations with the “landvR” package (Guillerme & Weisbecker, 2019). We performed UPGMA clustering analyses based on the morphological data (Figures S5 and S6) to contrast with the genetic‐based phylogenies of the populations (Hohnen et al., 2016; How et al., 2009; Woolley et al., 2015). We reconstructed the phenetic relationships based on the Euclidean distances among the population mean shapes (Figure S5) and among the specimens (Figure S6). We also estimated the morphological disparity (Procrustes variance) of island and mainland individuals and of each population and performed pairwise comparisons among these groups.

#### 2.4.2 Sexual dimorphism and allometry

To assess the degree to which shape variation in the sample was determined by sex differences, we computed the interaction term of size and sex on shape to evaluate whether both sexes had common allometric relationships. In addition, we corrected for allometric shape differences between sexes by extracting the residuals of allometry of each specimen and adding them to the consensus shape obtained from the GPA. This allowed us to make heat plots of sexual dimorphism of shape and size‐corrected shape.

In order to evaluate the influence of size on shape (allometry) in our dataset, we performed a Procrustes ANOVA to quantify the amount of shape variation influenced by the centroid size. We then plotted the centroid size versus the projected regression score of shape on size (Drake & Klingenberg, 2008). We used a homogeneity of slopes test to examine whether there was a common allometric pattern in all mainland populations plus the Groote Eylandt population.

#### 2.4.3 Association of shape variation with geography, climate, and size

We performed a partitioning analysis of cranial shape variation test for factors that may influence cranial shape, such as geographic distance among individuals or bioclimatic variables for the four mainland populations. For this, we used the *varpart* function in the *vegan* package for R version 2.5‐6 (Oksanen et al., 2018). Latitude and longitude coordinates of each locality corresponding to each specimen were transformed using a principal coordinates of neighborhood matrix (PCNM) (Borcard & Legendre, 2002) to avoid spatial autocorrelation. The PCNM method presents several advantages. It produces orthogonal (linearly independent) spatial variables over a much wider range of spatial scales (Pandolfi, Maiorino, & Sansalone, 2015; Sansalone, Kotsakis, & Piras, 2015). We retained the PCNM axes showing positive eigenvalues, then we checked for significance for the selected axes and these (*n* = 10) were the ones included in the analyses. Environmental and biogeophysical data for all specimens (elevation, distance to permanent water, primary productivity and vegetation type; annual mean temperature and annual precipitation) were obtained from the Atlas of Living Australia (www.ala.org.au) and WORLDCLIM (v. 2.0) (www.worldclim.org/bioclim), respectively. Those variables that had a clear effect on size and/or shape variation were included in the final model. Finally, we performed a redundancy analysis (RDA) on the partial and full models with 1,000 permutations, which includes the three factors (size, spatial data, and climatic data) that we hypothesized to explain the variation on cranial shape in our study system.

## 3 RESULTS

### 3.1 Morphological differences between populations

**Figure 1:**
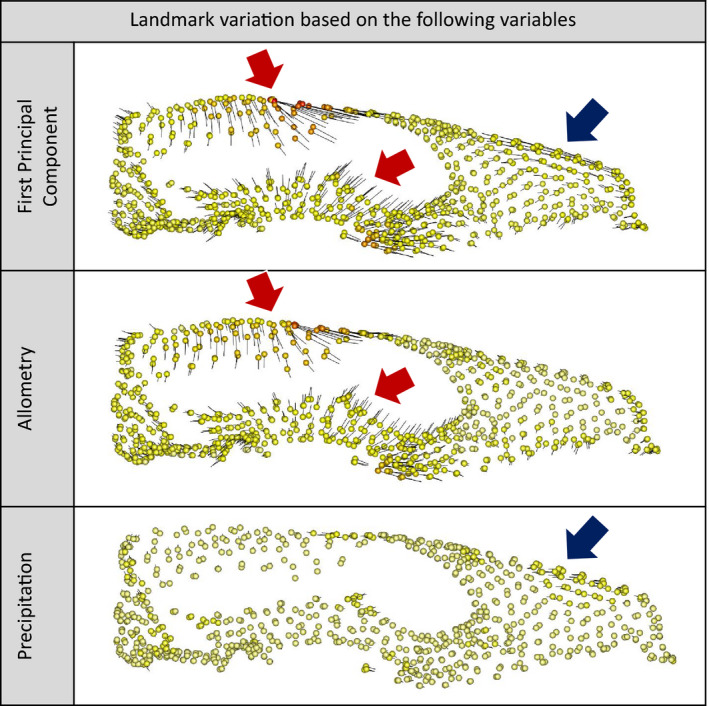
Shape changes associated with the First Principal Component (above), allometry (middle), and precipitation (below). Spheres are the landmarks used in this study. Warmer colours represent higher landmark variation between minimum and maximum estimated configurations based on the three linear predictors. Black vectors show direction and magnitude of shape variation. Red arrows indicate anatomical zones of major landmark variation associated with allometry. Blue arrow indicates anatomical zone of major landmark variation associated with precipitation

The principal component analysis reveals no visually obvious shape differentiation among populations along the two main axes of variation. The first two principal components together account for 35% of the total shape variation (PC1 = 24.58%, PC2 = 11.58%) (Figure S3). An example of shape changes along the first principal component axis with allometric shape changes put together with other unrelated shape changes is illustrated in Figure 1.

**Figure 2:**
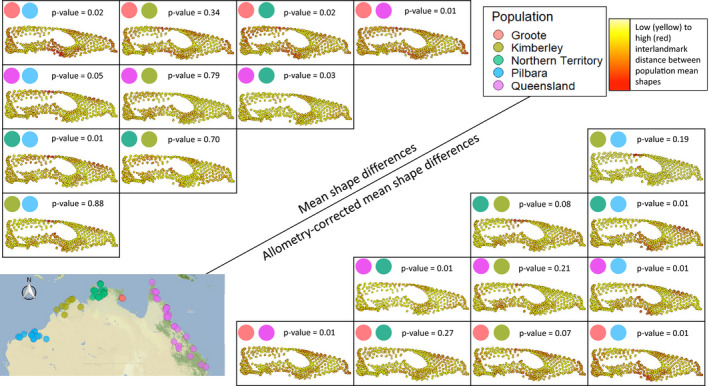
Pairwise comparisons between means of each population and visualization of interlandmark variation between populations mean shapes. Warmer colours represent higher landmark variation. Top left, comparisons between mean shapes of each population; bottom right, comparisons between size‐corrected mean shapes of each population. Map on bottom left shows all specimen locations used in this study. P values for pairwise comparisons are corrected with the Bonferroni method. Note that the colour range in this figure is calculated within the minimum and maximum inter‐landmark differences between population comparisons and is therefore not comparable to the colour range of Figure 1. Using the same colour range would mask the population differences depicted here. Also note that black vectors of direction and magnitude of variation (these are comparable to Figure 1) are barely visible because the shape differences are very small

Despite the low differentiation of populations within the PCA, the Procrustes ANOVA indicates that at least one of the five populations differs significantly in shape with low effect (Table 1). The post hoc pairwise comparisons between the shape means of each population reveal significant differences in shape among all populations (Figure 2). Intriguingly, the only sex‐biased population (Kimberley, which consisted mostly of males) shows no clear difference with respect to the remaining four populations. Groote Eylandt specimens show a generally narrower skull as revealed by the greater interlandmark distances in the zygomatic arches. The four mainland populations have shorter muzzles than the Groote specimens, as revealed by the shortening of the nasal and frontal areas. Northern Territory specimens display elongated frontal bones. Pilbara specimens exhibit an expansion of the braincase size and shorter muzzles relative to the rest of the skull. Pairwise comparisons among mainland populations (Table S3) revealed that only Groote Eylandt population was significantly different in shape disparity (Procrustes variance) relative to the mainland populations except Kimberley. Sexes (*d* = 0.0001, *Z* = 1.267, *p* = .123) and island versus mainland specimens (*d* = 0.0003, *Z* = 1.574, *p* = .083) did not reveal significant differences in shape disparity.

**Table 1:** ANOVA on predictors of size variation and Procrustes ANOVA on predictors of shape variation

Response variable	Predictor variable	Question	*df*	SS	*R* ^2^	*F*	Pr(>*F*)	Interpretation
Size	Population	Are populations different in size?	4	60,946	0.269	8.361	0.001	Clear effect
	Sex	Are sexes different in size?	1	48,372	0.21	23.9	<0.001	Clear effect
	Island/ Mainland	Are island and mainland individuals different in size?	1	30,700	0.125	14.15	<0.001	Clear effect
Shape	Population	Are populations different in shape?	4	0.015	0.121	3.125 (*F* critical value = 5.058)	0.001	Low effect size and unclear biological importance.
	Size	Is there allometry?	1	0.02	0.169	19.087	0.001	Clear effect, relatively high effect sizes
	Sex	Are sexes different in shape?	1	0.012	0.102	10.274	0.001	Clear effect
	Size: Sex	As there is sexual dimorphism and allometry, do sexes differ in allometric slopes?	1	0.001	0.013	1.429	0.1	No clear effect
	Size + Sex	Adjusting for size, are sexes different in shape?	1	0.005	0.045	5.116 (*F* critical value = 11.582)	0.001	Low effect sizes and low variance explained
	Size: Population	Do populations differ in allometric slopes?	4	0.004	0.036	1.133	0.215	No clear effect
	Size + Population	Adjusting for size, are populations different in shape?	4	0.013	0.131	3.419 (*F* critical value = 5.064)	0.001	Low effect size and unclear biological importance
	Island/ Mainland	Are island and mainland individuals different in shape?	1	0.003	0.025	2.518 (*F* critical value = 7.586)	0.007	Low effect sizes and low variance explained

Note

**Figure 3:**
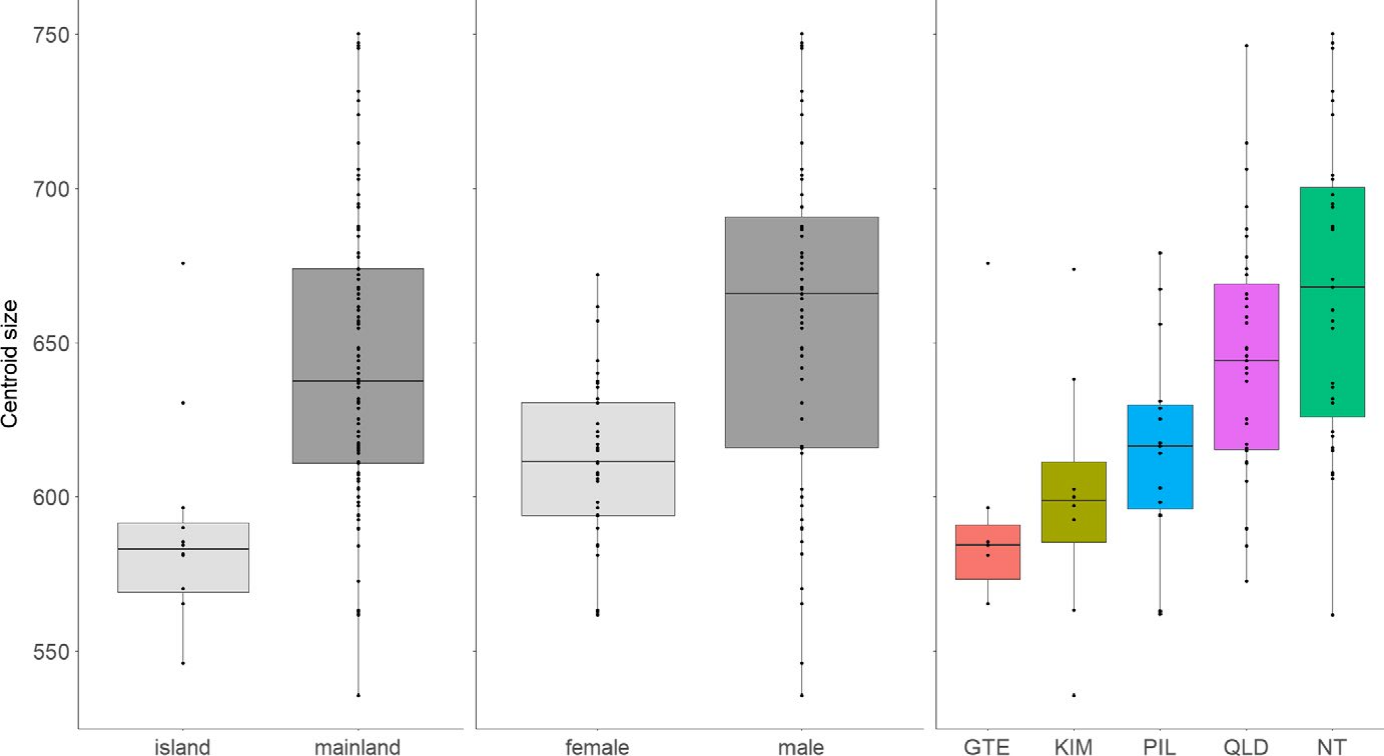
Box plots and dot plots of Centroid Size according to island/mainland, sex and population. Population abbreviations: GTE, Groote Eylandt, KIM, Kimberley, NT, Northern Territory, PIL, Pilbara, QLD, Queensland

*F* critical values provided where there is statistical lack of clarity. For direction of the effect of populations, sexes and island/mainland individuals on size variation, please refer to Figure 3.

### 3.2 Sexual dimorphism and allometry

We first confirmed that known sexual dimorphism in animal weight and skeletal measurements (Oakwood, 1997; Schmitt et al., 1989) are reflected in cranial size (Figure 3) and shape (Table 1; Figure S4). Size differences are significant between males and females, island and mainland populations and populations. Pairwise comparisons of size means between populations are shown in Table S2.

**Figure 4:**
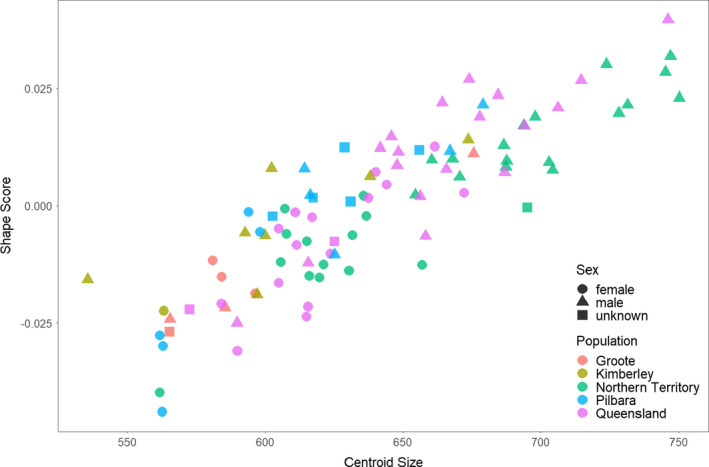
Allometry plot, centroid sizes (proxy for body size) versus shape scores obtained from the regression of shape on size (Drake & Klingenberg, 2008). Results of Homogeneity of Slopes Test for allometric slopes of populations are shown on Table 1

Females and males show no significant difference between allometric slopes (Table 1), such that small males and large females overlap on the allometric slope (see Figure 4). Most of the sex‐related differences in shape are due to sex differences in size. We found statistically significant but low effects of sex‐related differences in allometry‐corrected shape (Table 1 and Figure S4). In other words, although there is some non‐size‐related variation between the sexes, small males and large females are similarly shaped according to their common size. We therefore included individuals of both sexes in our analyses of population differences.

In the full dataset, and among all variables tested, size manifests as the strongest determinant of shape variation in northern quolls, accounting for 16.9% of the total shape variation. A homogeneity of slopes test suggests no significant differences among allometric slopes of each population (Table 1 and Figure 4), meaning that the hypothesis of populations following the same allometric slope is not rejected. Allometry‐corrected shape analysis also reveals the shape differences between populations; Procrustes ANOVA performed on the residual shapes of allometry revealed significant differences between populations but with low effect sizes (Table 1). Pairwise comparisons between these size‐corrected shapes show similar significant differences among populations (Figure 2). Thus, allometry does not appear to play a full role in differentiating the shape of populations.

We visualized the functional implications of shape divergence among the northern quolls, by examining the displacement between landmark configurations according to size and sex. The landmark displacement predicted by allometry identifies two main regions of variation: Larger skulls tend to have overall smaller braincases relative to the rest of the skull, a larger sagittal crest, a more anteriorly positioned masseteric scar and associated dorsally oriented zygomatic arch. The minor differences between sexes include males with larger sagittal crests, smaller braincases, shorter nasals, and dorsally oriented zygomatic arches.

### 3.3 Association of shape variation with geography, climate, and size

Significant shape differences were observed along both latitudinal and longitudinal gradients on mainland specimens (for effect sizes and significance levels, refer to Table 2). Size is significantly different only along the latitudinal gradient but not the longitudinal gradient. Temperature and precipitation have a significant, but small, effect on shape. Size differences are only explained by precipitation and not by temperature. Other biogeophysical and environmental variables tested did not contribute significantly to size or shape variation (Table 2).

**Table 2:** Analysis of Variance on sources of size and shape variation of mainland specimens

	*df*	Size				Shape			
		SS	*R* ^2^	*F*	PR(>*F*)	SS	*R* ^2^	*F*	PR(>*F*)
Latitude	1	18,618	0.092	8.831	0.004	0.005	0.044	4.051	0.001
Longitude	1	5,666	0.028	2.51	0.117	0.004	0.034	3.023	0.001
Precipitation	1	17,441	0.086	8.22	0.005	0.004	0.038	3.406	0.001
Temperature	1	159	0.001	0.069	0.794	0.003	0.023	2.006	0.026
Elevation	1	1,433	0.007	0.622	0.433	0.001	0.01	0.898	0.527
Distance to permanent water	1	1,064	0.005	0.461	0.499	0.001	0.011	0.997	0.401
Primary productivity	1	2,146	0.01	0.934	0.337	0.001	0.013	1.139	0.289
Vegetation type	1	25,061	0.124	1.243	0.282	0.013	0.116	1.157	0.162

**Figure 5:**
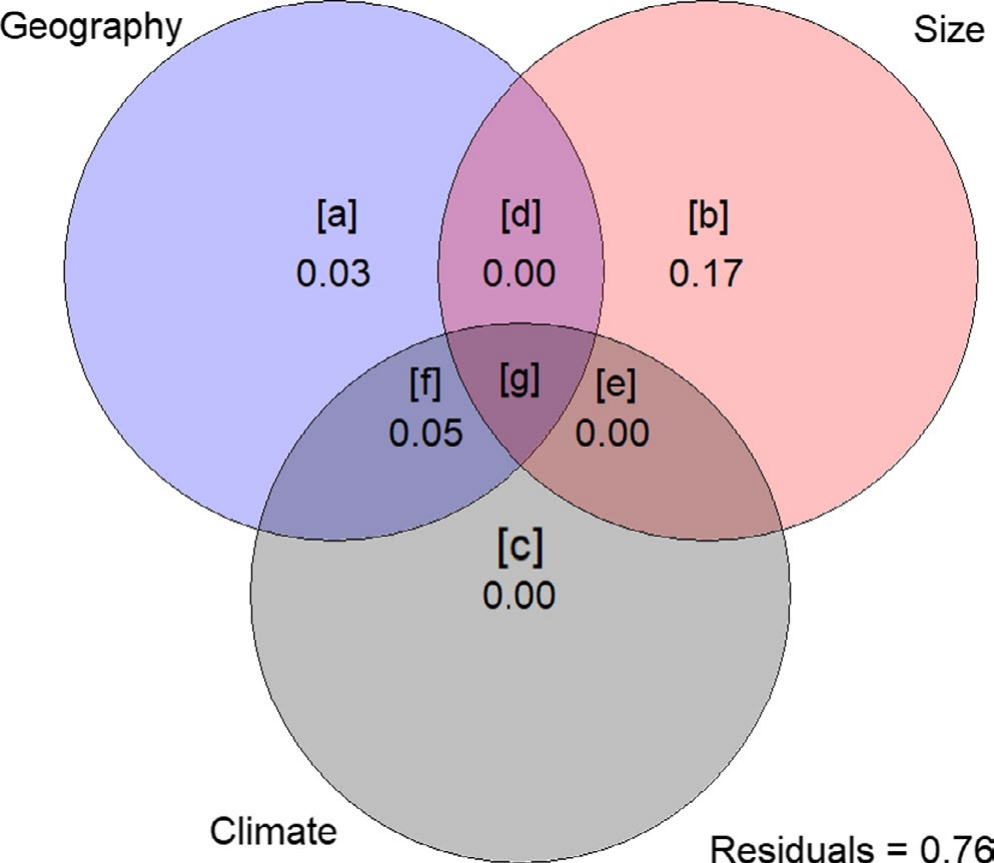
Schematic representation of the variation partitioning analysis (VARPART), which included effect of geography, size and combined climatic variables (precipitation and temperature) on cranial shape of mainland specimens. The values shown in the diagram represent the individual fractions for each set. The outer numbers are the adjusted *R* ^2^ values of pure geography [a], pure size [b] and pure climate [c] and the inner values are the adjusted R‐squared values of the interaction of the corresponding explanatory variables. The individual fraction for the interaction of all three variables [g] is negligible and not shown. The amount of unexplained shape by this model is depicted by the residuals (76%). Circle sizes are schematic and do not represent the amount of shape explained by the model

We dissected the influence of size, geography and climate (precipitation + temperature) with a variation partitioning analysis (Figure 5). The full model including all parameters ([a + b + c + d + e + f + g] on Figure 5) shows a significant effect of these three factors on cranial shape variation (*F*_13,75_ = 3.151, adjusted *R* ^2^ = 0.24, *p* = .001). Climatic variables alone [c] do not explain any of the variation (*F*_2,75_ = 1.18, adjusted *R* ^2^ = 0.004, *p* = .189); however, they contribute to the model when geography is considered jointly [f] (*F*_2,86_ = 2.856, adjusted *R* ^2^ = 0.05, *p* = .002). Pure geographic distances [a] explain 3% (adjusted *R* ^2^ = 0.034) of the shape variation (*F*_10,75_ = 1.388, *p* = .001). Finally, in accordance with our predictions, size alone [b] contributes mostly to the model by accounting for 17% of the total cranial shape variation (*F*_1,75_ = 17.482, adjusted *R* ^2^ = 0.165, *p* = .001).

## 4 DISCUSSION

We expected that the biogeographic adaptive and genetic divergences between northern quolls should be evident across their 5000‐kilometer longitudinal range. Our aim was to improve our understanding on whether the species represent morphological or functional conservation units, which could be used alongside population genetic approaches in the conservation management of the species. Surprisingly, however, we found little structure in northern quoll shape variation (~76% of shape variation remains unexplained) and no strong evidence that any of the populations have evolved into discrete, possibly locally adapted, morphotypes. In particular, population differences have low effect sizes and explain less variation on shape than size. This appears to be a one‐to‐many mapping case in which similarly sized individuals from opposite ends of the biogeographic distribution and under distinct climatic conditions with likely different functional constraints are likely to be similar in shape. It also seems that most variation is evenly distributed within each of the populations, such that more localized western populations appear just as disparate in shape as individuals across the length of the eastern Queensland seaboard.

There is some limited support for the hypothesis that developmental constraints might reduce the adaptability of northern quoll skulls, as size explains most shape variation and the populations seem to differ in body size. Given the low amount of variation (~16%) that size explains, and the broad overlap of populations in size and shape, any such constraint is unlikely to be strong. However, there is an intriguing indication that at least some of the larger‐scale evolutionary association between skull shape and size among marsupials may be visible at the within‐species level. This is contrary to findings in other marsupials where feeding behaviors clearly influenced craniofacial morphology (Mitchell, Sherratt, Sansalone, et al., 2018; Weisbecker et al., 2019), and might represent one of several ways to shape morphological traits within the species.

Shape appears to have not been affected by population identity, even when size is taken into account. This might suggest a stochastic, possibly heritable, shallow divergence between populations which, however, does not appear to reflect local adaptation. These effects also demonstrate the ability of skull shape to vary independently of size based on genetic factors related to sex or population, again contradicting the developmental constraints hypothesis. Thus, the population divergences do not appear to coincide with adaptive morphological differentiations. This provides an indication that genetic fitness benefits of outbreeding populations (Cardoso et al., 2009; Kelly & Phillips, 2019) would not risk any adverse effects due to differential local adaptation, although of course this would need to be further investigated based on nonmorphological (behavioral or physiological) traits. However, these assumptions need to be interpreted carefully because the low effect sizes of population identity on shape variation indicate a very low biological impact on cranial shape of individuals (see also Weisbecker et al., 2019).

It is possible that much of the variation in the northern quoll skull derives from a remodeling process based on individual uses of the skull. Perhaps, the best example of this is the sagittal crest, which varies widely in length among northern quoll individuals. It is common for mammals—particularly males—to display larger sagittal crests with age (Flores, Giannini, & Abdala, 2006), but this is a purely behavioral consequence of the pulling action of the temporalis muscle (Washburn, 1947). Similarly, bone remodeling during an individual's lifetime has been suggested for the zygomatic arch of mammals (Abdala & Giannini, 2000; Ravosa, 1991), and is also suspected in wombats and kangaroos (Mitchell, Sherratt, Ledogar, et al., 2018; Mitchell, Sherratt, Sansalone, et al., 2018; Weisbecker et al., 2019). It is therefore possible that the emphasis of shape variation on the masticatory apparatus, found along PC1 and the allometric pattern, does not arise from heritable adaptation. Rather, they might derive from highly generalist masticatory behaviors and possibly the “mating bite” (Braithwaite & Griffiths, 1994; Oakwood, 2000) of the larger males. The nonallometric main shape variation found along PC1 (shortening of the muzzle) could be explained by differences in precipitation (proxy for dietary behaviors) (blue arrow in Figure 1). To a limited extent due to the small effect, this size‐corrected main shape variation could also be explained by nonallometric sexual differences (Figure S4), consistent with the weak tendency of males to have shorter muzzles than females, related to higher bite forces (Wroe & Milne, 2007).

In line with the size component being the strongest determinant of shape, the “island rule” (Flannery, 2002; Foster, 1964; Lomolino, 1985) appears to be supported by our results, with smaller body size in island individuals, which also accords with the observations of Hohnen et al. (2016) on northern quolls. Despite the close genetic connection between the Northern Territory and Groote Eylandt populations (Hohnen et al., 2016; Woolley et al., 2015), the Groote Eylandt population is most differentiated from all other mainland populations. This was also revealed in the cluster analyses, supporting the high genetic erosion observed by Cardoso et al. (2009) over 8,000 years of isolation from the mainland (Woinarski et al., 1999). The Groote Eylandt morphology reveals more compacted zygomatic arches combined with longer muzzles, which are changes associated along the allometric relationship and possibly reveal evolutionary shape changes tightly constrained by body size.

The genetic proximity of the Northern Territory and Queensland populations relative to the Pilbara and Kimberley populations (Hohnen et al., 2016; How et al., 2009; Woolley et al., 2015) appears to parallel our clustering analyses on population mean shapes. However, the unsubstantial morphological differentiation and very short branches limit the interpretation of the cluster analysis.

Aside from sexual and allometric shape variation, slight rearrangements of the zygomatic arch and the muzzle appear to provide some diffuse distinction between northern quoll populations. This variation is consistent with well‐known evolutionary adaptations to changes in mastication (Meloro, 2011; Mitchell, Sherratt, Sansalone, et al., 2018; Mitchell & Wroe, 2019; Weisbecker et al., 2019). The variation in muzzle length and zygomatic arch placement among some populations may be adaptations to a particular diet or feeding habit within each population. For instance, drier environments such as the Pilbara or Groote Eylandt show a shortening in the muzzle, when compared to wet environments, such as Northern Territory or Queensland. Shorter faces are known to imply greater bite forces, and thus might indicate the mastication of tougher foods (Wroe & Milne, 2007). However, although precipitation is a main predictor of quoll diets (Fancourt et al., 2015), this variable explains little variation in shape. Whether the shortening of the muzzle is truly an adaptive effect requires further research due to the lack of association between climatic factors and shape, and the abovementioned small effect sizes and extensive overlap in shape between populations. This provides a good starting point for discussion and future investigations to identify whether the differences among populations are or not a decisive factor for the individuals’ survival, and rather originate from potentially nonadaptive factors such as genetic drift or individual variation in feeding (Weisbecker et al., 2019) between populations.

An apparent limitation of our variation partitioning analyses is the high levels of unexplained variation. While these are reasonably common in geometric morphometrics, they are also generally not expected (Cardini, Filho, Polly, & Elton, 2010; Cardini, Jansson, & Elton, 2007; Hendges, Bubadué, & Cáceres, 2016; Pandolfi et al., 2015). Where unexplained variation is large, it is commonly presumed that other, unknown variables are responsible for this variation. This may also be the case in our analysis; alternatively, the effect of factors not measured for this study, such as genetic, physiological, or behavioral traits, could contribute to this proportion of unexplained variation.

The geometric morphometric analyses of the northern quoll skull add useful, quantitative, phenomic data to assessments of variation across the distribution of an endangered marsupial. The overarching find of low morphological differentiation, and very high levels of unexplained variation, has two important implications. First, it suggests that individuals of different populations are not locally adapted to the point where a separation of population phenomes is indicated (although it needs to be investigated whether there might be behavioral or physiological reasons to do so). On the other hand, the lack of differentiation across the diversity of biomes, climatic conditions, or populations is a concern because it suggests a low adaptability of the species to environmental change. The concentration of shape variation in the masticatory apparatus suggests individual plasticity is a major response mechanism in the determination of northern quoll skull shape, suggesting that there is little scope for larger‐scale, heritable variation within the species. A similar pattern of potentially high within‐species plasticity in the masticatory apparatus has also been suggested for the living wombat species as well as kangaroos; together, the concerning suggestion is that marsupial mammals might have a scope for individual plasticity, but not evolve specific adaptations within short time spans. Further research should be directed into identifying the scope of shape variation in other threatened marsupials, investigating other climatic variables or patterns as predictors, and adding biomechanical and developmental studies to further understand the variation that exists in this clade; in addition, a comparison with ecologically similar placental species would be useful to identify whether marsupials show less intrinsic capacity of shape variation than placental mammals.

## CONFLICT OF INTEREST

The authors declare no conflict of interest.

## AUTHOR CONTRIBUTION

**Pietro Francesco Viacava:** Conceptualization (lead); Data curation (lead); Formal analysis (lead); Investigation (lead); Methodology (lead); Project administration (equal); Software (lead); Validation (lead); Visualization (lead); Writing‐original draft (lead); Writing‐review & editing (equal). **Simone P. Blomberg:** Formal analysis (supporting); Methodology (supporting); Software (supporting); Supervision (equal); Validation (supporting); Writing‐review & editing (equal). **Gabriele Sansalone:** Formal analysis (supporting); Methodology (supporting); Software (supporting); Validation (supporting); Writing‐review & editing (equal). **Matthew J Phillips:** Conceptualization (equal); Funding acquisition (lead); Resources (equal); Supervision (equal); Validation (equal); Writing‐review & editing (equal). **Thomas Guillerme:** Data curation (supporting); Formal analysis (supporting); Methodology (supporting); Resources (supporting); Software (supporting); Writing‐review & editing (equal). **Skye F. Cameron:** Data curation (supporting); Investigation (supporting); Resources (supporting); Writing‐review & editing (supporting). **R. S. Wilson:** Data curation (supporting); Investigation (supporting); Resources (supporting); Supervision (supporting); Writing‐review & editing (supporting). **Vera Weisbecker:** Conceptualization (lead); Data curation (supporting); Formal analysis (equal); Funding acquisition (lead); Investigation (supporting); Methodology (supporting); Project administration (equal); Resources (lead); Software (supporting); Supervision (lead); Validation (lead); Visualization (supporting); Writing‐original draft (lead); Writing‐review & editing (equal).

### Open Research Badges

This article has been awarded Open Materials, Open Data Badges. All materials and data are publicly accessible via the Open Science Framework at https://datadryad.org/stash/share/OTViI4GO1rmqF0vcW6mX9n1A1VnfymzSbtYTEQWXvoA, https://github.com/pietroviama/Viacavaetal_Dhallucatus, https://www.morphosource.org/Detail/ProjectDetail/Show/project_id/834

## Supporting information

Appendix S1

SupInfo1

SupInfo2

SupInfo3

## Data Availability

Data are publicly available in Dryad. R code is also publicly available in GitHub. 3D models can be publicly accessed through MorphoSource. Dryad: https://datadryad.org/stash/share/OTViI4GO1rmqF0vcW6mX9n1A1VnfymzSbtYTEQWXvoA. Github: https://github.com/pietroviama/Viacavaetal_Dhallucatus. https://www.morphosource.org/Detail/ProjectDetail/Show/project_id/834
