# Supplementary material for: Skull shape of a widely distributed, endangered marsupial reveals little evidence of local adaptation between fragmented populations
Source: Ecol Evol. 2020 Aug 18;10(18):9707–20. doi: 10.1002/ece3.6593 (PMC7520215; doi:10.1002/ece3.6593)
Supplement: Supplementary file 1 — Appendix S1 [file ECE3-10-9707-s001.docx]

**ix. SUPPORTING INFORMATION**
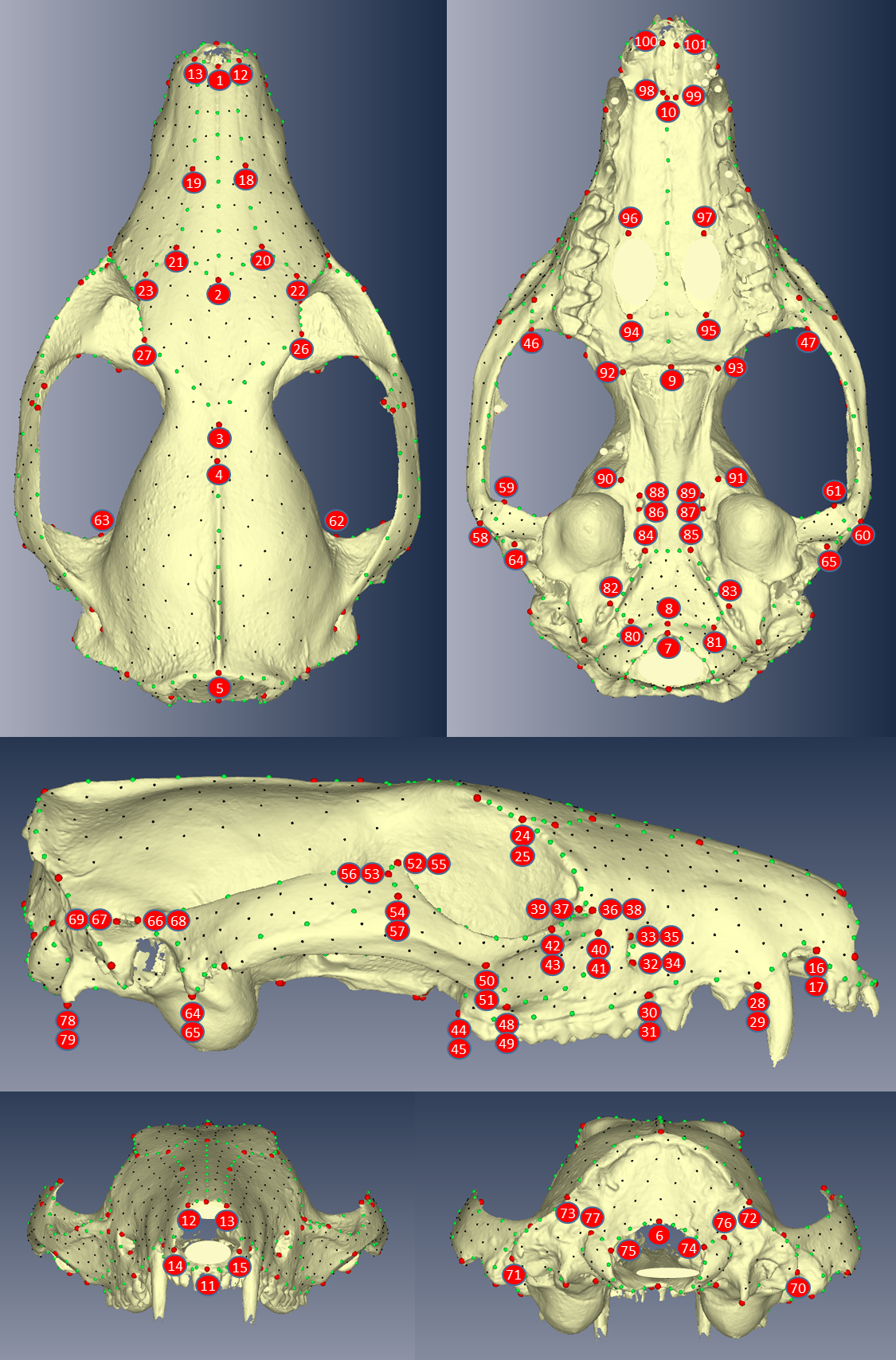


Figure S1: Template used in this study, containing 101 fixed landmarks (in red), 271 curve semilandmarks (in green) and 528 surface semilandmarks (in black). For landmarks definitions, please refer to Supplementary Table 1.

Table S1: Anatomical definitions of fixed landmarks, curves and patches.

| **Quantity** | **Fixed landmarks** | **Curves (number of semilandmarks)** | **Patches (number of semilandmarks)** |
| --- | --- | --- | --- |
| 1 | LM Nasal, anterior, midline | LM1 to LM12, anterior nasal (1) | SUR Nasal right (9) |
| 2 | LM Nasal-frontal suture, midline | LM12 to LM14, anterior premaxillary (3) | SUR Nasal left (9) |
| 3 | LM Intersection of temporal lines, midline | LM14 to LM11, anterior edge of premaxillary (3) | SUR Frontal (35) |
| 4 | LM frontal-parietal suture, midline | LM11 to LM15, anterior edge of premaxillary (3) | SUR Right Premaxillary-maxillary (72) |
| 5 | LM External occipital protuberance, nuchal crest, midline | LM15 to LM13, anterior premaxillary (3) | SUR Left premaxillary-maxillary (72) |
| 6 | LM Dorsal lip of foramen magnum, midline | LM13 to LM1, anterior nasal (1) | SUR right anterior jugal (18) |
| 7 | LM Ventral lip of foramen magnum, midline | LM1 to LM2, midline, nasal suture (8) | SUR left anterior jugal (18) |
| 8 | LM Midline point on the concave transition between the occipital condyle and the basioccipital | LM12 to LM18, nasal-premaxillary suture (3) | SUR right posterior jugal (26) |
| 9 | LM Palatine, posterior, midline | LM13 to LM19, nasal premaxillary suture (3) | SUR left posterior jugal (26) |
| 10 | LM Medial point of the most posterior side of the incisive foramina | LM18 to LM20, nasal-maxillary suture (3) | SUR right squamosal (22) |
| 11 | LM Midline point on the premaxilla at the inferior tip of the bony septum between the upper central incisors | LM19 to LM21, nasal-maxillary suture (3) | SUR left squamosal (22) |
| 12 | LM R Nasal-premaxillary suture, anterior (on nasal) | LM11 to LM16, anterio-ventral edge of premaxillary (4) | SUR right glenoid fossa (9) |
| 13 | LM L Nasal-premaxillary suture, anterior (on nasal) | LM11 to LM17, anterio-ventral edge of premaxillary (4) | SUR left glenoid fossa (9) |
| 14 | LM R Maximum concavity on anterior of premaxillary | LM20 to LM 2, nasal-frontal suture (3) | SUR basioccipital (25) |
| 15 | LM L Maximum concavity on anterior of premaxillary | LM21 to LM2, nasal-frontal suture (3) | SUR left and right occipital condyles (24) |
| 16 | LM R Premaxillary-maxillary suture, anterior, lateral end | LM20 to LM22, frontal-maxillary suture (2) | SUR posterior supraoccipital-exoccipital-petrosal edge (48) |
| 17 | LM L Premaxillary-maxillary suture, anterior, lateral end | LM21 to LM23, frontal-maxillary suture (2) | SUR right braincase (42) |
| 18 | LM R Premaxillary-maxillary suture, posterior end in dorsal view | LM22 to LM24, frontal-lacrimal suture (2) | SUR left braincase (42) |
| 19 | LM L Premaxillary-maxillary suture, posterior end in dorsal view | LM23 to LM25, frontal-lacrimal suture (2) | **18 patches (528 semilandmarks)** |
| 20 | LM R Frontal-maxillary-nasal intersection | LM24 to LM26, edge of postorbital process of frontal (3) |  |
| 21 | LM L Frontal-maxillary-nasal intersection | LM25 to LM27, edge of postorbital process of frontal (3) |  |
| 22 | LM R Frontal-maxillary-lacrimal intersection | LM24 to LM37, postorbital process, edge of lacrimal (5) |  |
| 23 | LM L Frontal-maxillary-lacrimal intersection | LM37 to LM42, edge of lacrimal (3) |  |
| 24 | LM R Frontolacrimal suture, on postorbital process, dorsal | LM25 to LM39, postorbital process, edge of lacrimal (5) |  |
| 25 | LM L Frontolacrimal suture, on postorbital process, dorsal | LM39 to LM43, edge of lacrimal (3) |  |
| 26 | LM R Zygomatic process of frontal, maximum convexity | LM26 to LM3, temporal line (4) |  |
| 27 | LM L Zygomatic process of frontal, maximum convexity | LM27 to LM3, temporal line (4) |  |
| 28 | LM R Most posterio-distal point in upper canine neck | LM16 to LM28, ventro-distal maxillary edge (3) |  |
| 29 | LM L Most posterio-distal point in upper canine neck | LM28 to LM30, ventro-distal maxillary edge (2) |  |
| 30 | LM R Most antero-distal point in upper first molar neck | LM30 to LM44, ventro-distal maxillardy edge (4) |  |
| 31 | LM L Most antero-distal point in upper first molar neck | LM44 to LM46, along most posterior ridge of maxillary (2) |  |
| 32 | LM R Most ventral point of infraorbital foramen | LM32 to LM 33, distal width of infraorbital foramen (1) |  |
| 33 | LM R Most dorsal point of infraorbital foramen | LM33 to LM 32, proximal width of infraorbital foramen (1) |  |
| 34 | LM L Most ventral point of infraorbital foramen | LM36 to LM37, ventral width of antero-ventral lacrimal foramen (1) |  |
| 35 | LM L Most dorsal point of infraorbital foramen | LM37 to LM36, dorsal width of antero-ventral lacrimal foramen (1) |  |
| 36 | LM R Most anterior point in antero-ventral lacrimal foramen | LM17 to LM29, ventro-distal maxillary edge (3) |  |
| 37 | LM R Most posterior point in antero-ventral lacrimal foramen | LM29 to LM31, ventro-distal maxillary edge (2) |  |
| 38 | LM L Most anterior point in antero-ventral lacrimal foramen | LM31 to LM45, ventro-distal maxillardy edge (4) |  |
| 39 | LM L Most posterior point in antero-ventral lacrimal foramen | LM45 to LM47, along most posterior ridge of maxillary (2) |  |
| 40 | LM R Maxillary-lacrimal-jugal intersection | LM34 to LM 35, distal width of infraorbital foramen (1) |  |
| 41 | LM L Maxillary-lacrimal-jugal intersection | LM35 to LM 34, proximal width of infraorbital foramen (1) |  |
| 42 | LM R Most dorsal point in lacrimal-jugal suture (zygomatic arch) | LM38 to LM39, ventral width of antero-ventral lacrimal foramen (1) |  |
| 43 | LM L Most dorsal point in lacrimal-jugal suture (zygomatic arch) | LM39 to LM38, dorsal width of antero-ventral lacrimal foramen (1) |  |
| 44 | LM R Most posterior point in fourth molar neck | LM40 to LM42, lacrimal-jugal suture (2) |  |
| 45 | LM L Most posterior point in fourth molar neck | LM40 to LM48, jugal-maxillary suture (5) |  |
| 46 | LM R Most posterior point in the jugal-maxillary suture (zygomatic arch) | LM48 to LM 50, along jugal ridge (2) |  |
| 47 | LM L Most posterior point in the jugal-maxillary suture (zygomatic arch) | LM48 to LM46, jugal-maxillary suture (2) |  |
| 48 | LM R Most lateral point in the jugular-maxillary suture | LM41 to LM43, lacrimal-jugal suture (2) |  |
| 49 | LM L Most lateral point in the jugular-maxillary suture | LM41 to LM49, jugal-maxillary suture (5) |  |
| 50 | LM R Most posterio-dorsal point in the muscular depression (zygomaticus and levator labii) in the jugal | LM49 to LM 51, along jugal ridge (2) |  |
| 51 | LM L Most posterio-dorsal point in the muscular depression (zygomaticus and levator labii) in the jugal | LM49 to LM47, jugal-maxillary suture (2) |  |
| 52 | LM R Most dorsal point in jugal | LM3 to LM4, midline, sagittal crest (1) |  |
| 53 | LM R Most dorsal point in the jugal-squamosal suture | LM4 to LM5, midline, sagittal crest (5) |  |
| 54 | LM R Most anterior point in the jugal squamosal suture | LM42 to LM52, dorsal, frontal process of the jugal (5) |  |
| 55 | LM L Most dorsal point in jugal | LM52 to LM53, dorsal, frontal process of the jugal (1) |  |
| 56 | LM L Most dorsal point in the jugal-squamosal suture | LM53 to LM66, squamosal, most dorsal edge in zygomatic arch (4) |  |
| 57 | LM L Most anterior point in the jugal squamosal suture | LM53 to LM54, jugal-squamosal suture (1) |  |
| 58 | LM R Most posterio-distal point in jugal-squamosal suture | LM54 to LM58, jugal-squamosal suture (3) |  |
| 59 | LM R Most posterio-mesial point in the jugal-squamosal suture | LM46 to LM59, jugal, most ventral edge in zygomatic arch (5) |  |
| 60 | LM L Most posterio-distal point in jugal-squamosal suture | LM59 to LM62, anterior edge of glenoid fossa (2) |  |
| 61 | LM L Most posterio-mesial point in the jugal-squamosal suture | LM58 to LM59, squamosal-jugal suture (1) |  |
| 62 | LM R Maximum concavity in the posterio-ventral side of zygomatic arch, in alisphenoid | LM58 to LM64, squamosal distal edge of the glenoid fossa (3) |  |
| 63 | LM L Maximum concavity in the posterio-ventral side of zygomatic arch, in alisphenoid | LM64 to LM66, distal edge and shortest distance (3) |  |
| 64 | LM R Postglenoid process, ventral | LM43 to LM55, dorsal, frontal process of the jugal (5) |  |
| 65 | LM L Postglenoid process, ventral | LM55 to LM56, dorsal, frontal process of the jugal (1) |  |
| 66 | LM R Suprameatal foramen, anterior | LM56 to LM68, squamosal, most dorsal edge in zygomatic arch (4) |  |
| 67 | LM R Suprameatal foramen, posterior | LM56 to LM57, jugal-squamosal suture (1) |  |
| 68 | LM L Suprameatal foramen, anterior | LM57 to LM60, jugal-squamosal suture (3) |  |
| 69 | LM L Suprameatal foramen, posterior | LM47 to LM61, jugal, most ventral edge in zygomatic arch (5) |  |
| 70 | LM R Posttympanic process, maximum distal convexity | LM61 to LM63, anterior edge of glenoid fossa (2) |  |
| 71 | LM L Posttympanic process, maximum distal convexity | LM60 to LM61, squamosal-jugal suture (1) |  |
| 72 | LM R Supraoccipital-petrosal-squamosal intersection on nuchal crest | LM60 to LM65, squamosal distal edge of the glenoid fossa (3) |  |
| 73 | LM L Supraoccipital-petrosal-squamosal intersection on nuchal crest | LM65 to LM68, distal edge and shortest distance (3) |  |
| 74 | LM R Maximum concavity in distal of foramen magnum (most dorso-mesial point in occipital condyle) | LM5 to LM72, edge of nuchal crest (4) |  |
| 75 | LM L Maximum concavity in distal of foramen magnum (most dorso-mesial point in occipital condyle) | LM5 to LM73, edge of nuchal crest (4) |  |
| 76 | LM R Most dorsal point of the occipital condyle | LM72 to LM70, edge of nuchal crest (4) |  |
| 77 | LM L Most dorsal point of the occipital condyle | LM73 to LM71, edge of nuchal crest (4) |  |
| 78 | LM R Paracondylar process of the exoccipital, ventral | LM70 to LM78, edge of nuchal crest and shortest distance to paracondylar process (3) |  |
| 79 | LM L Paracondylar process of the exoccipital, ventral | LM71 to LM79, edge of nuchal crest and shortest distance to paracondylar process (3) |  |
| 80 | LM R Most mesial point in hypoglossal foramen | LM6 to LM74, edge of foramen magnum (3) |  |
| 81 | LM L Most mesial point in hypoglossal foramen | LM74 to LM7, edge fo foramen magnum (5) |  |
| 82 | LM R Most anterior point in jugular foramen | LM6 to LM75, edge of foramen magnum (3) |  |
| 83 | LM L Most anterior point in jugular foramen | LM75 to LM7, edge of foramen magnum (5) |  |
| 84 | LM R Most anterior point of the basioccipital | LM74 to LM76, edge of occipital condyle (1) |  |
| 85 | LM L Most anterior point of the basioccipital | LM75 to LM77, edge of occipital condyle (1) |  |
| 86 | LM R Most posterior point in foramen ovale | LM76 to LM8, edge of occipital condyle (5) |  |
| 87 | LM L Most posterior point in foramen ovale | LM77 to LM8, edge of occipital condyle (5) |  |
| 88 | LM R Most anterior point in foramen ovale | LM80 to LM82, shortest distance (2) |  |
| 89 | LM L Most anterior point in foramen ovale | LM81 to LM83, shortest distance (2) |  |
| 90 | LM R Most posterior point of the pterygoid hamulus | LM82 to LM84, petrosal-basioccipital ridge (3) |  |
| 91 | LM L Most posterior point of the pterygoid hamulus | LM83 to LM85, petrosal-basioccipital ridge (3) |  |
| 92 | LM R Postpalatine torus, distal | LM84 to LM85, basioccipital-basisphenoid suture (3) |  |
| 93 | LM L Postpalatine torus, distal | LM9 to LM10, midline (8) |  |
| 94 | LM R Major palatine foramen, posterior | **93 curves (271 semilandmarks)** |  |
| 95 | LM L Major palatine foramen, posterior |  |  |
| 96 | LM R Major palatine foramen, anterior |  |  |
| 97 | LM L Major palatine foramen, anterior |  |  |
| 98 | LM R Incisive foramen, posterior |  |  |
| 99 | LM L Incisive foramen, posterior |  |  |
| 100 | LM R Incisive foramen, anterior |  |  |
| 101 | LM L Incisive foramen, anterior |  |  |
|  | **101 Fixed landmarks** |  |  |


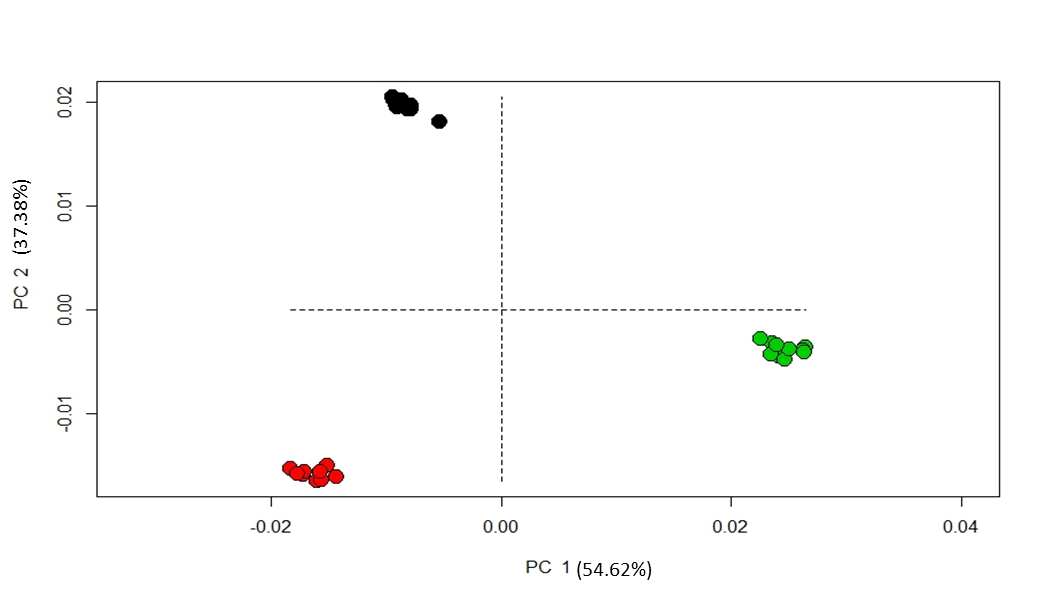


Figure S2: Repeatability test. Principal Component Analysis on ten repetitions of three morphologically close specimens.


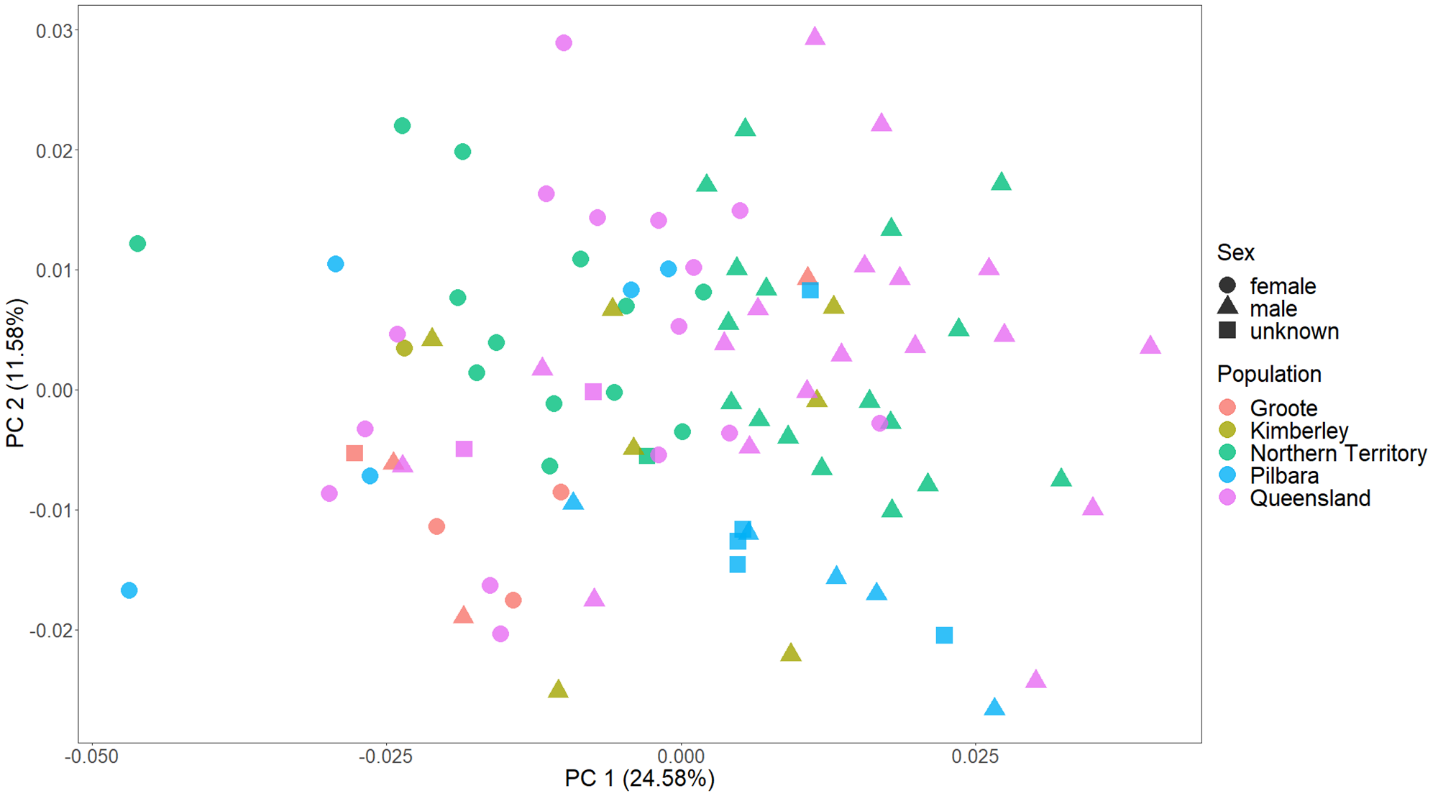


Figure S3: Principal Component Analysis on all specimens.


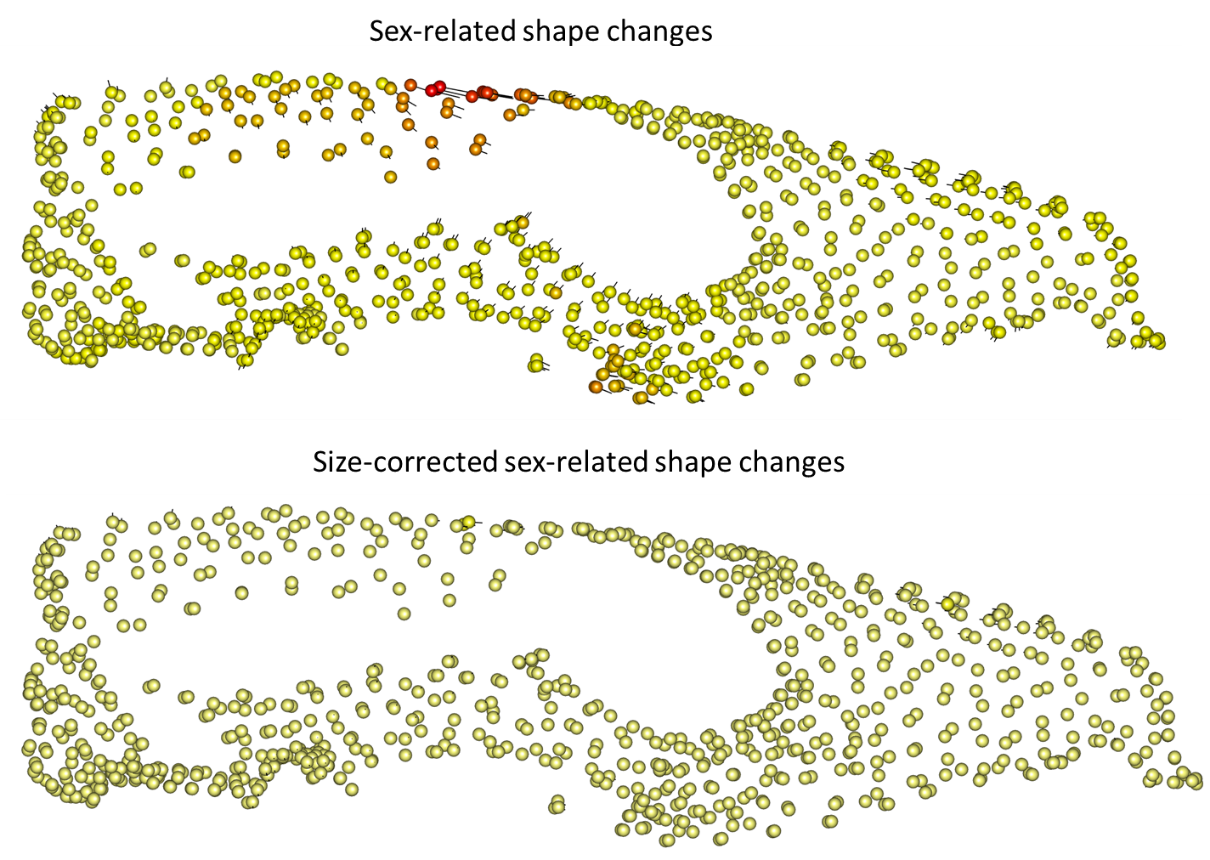


Figure S4: Heat plot on shape differences between estimated mean configurations of females and males. Warmer colours represent higher landmark variation between mean shapes.

Table S2: Pairwise comparisons of size means between populations with resampling procedure of 1000 random permutations. Values shown in bottom left are p-values associated with pairwise differences. In top right, the level of significance.

|  | Groote | Kimberley | Northern Territory | Pilbara | Queensland |
| --- | --- | --- | --- | --- | --- |
| Groote |  | ns | ** | ns | * |
| Kimberley | 0.782 |  | ** | ns | * |
| Northern Territory | 0.001 | 0.002 |  | ** | ns |
| Pilbara | 0.351 | 0.505 | 0.002 |  | * |
| Queensland | 0.014 | 0.018 | 0.052 | 0.049 |  |

Table S3: Pairwise comparisons of Procrustes shape disparities (variances) between mainland populations with resampling procedure of 1000 random permutations. Values shown in bottom left are p-values associated with pairwise differences. In top right, the level of significance.

|  | Groote | Kimberley | Northern Territory | Pilbara | Queensland |
| --- | --- | --- | --- | --- | --- |
| Groote |  | ns | * | * | * |
| Kimberley | 0.138 |  | ns | ns | ns |
| Northern Territory | 0.018 | 0.483 |  | ns | ns |
| Pilbara | 0.032 | 0.518 | 0.985 |  | ns |
| Queensland | 0.023 | 0.550 | 0.868 | 0.899 |  |


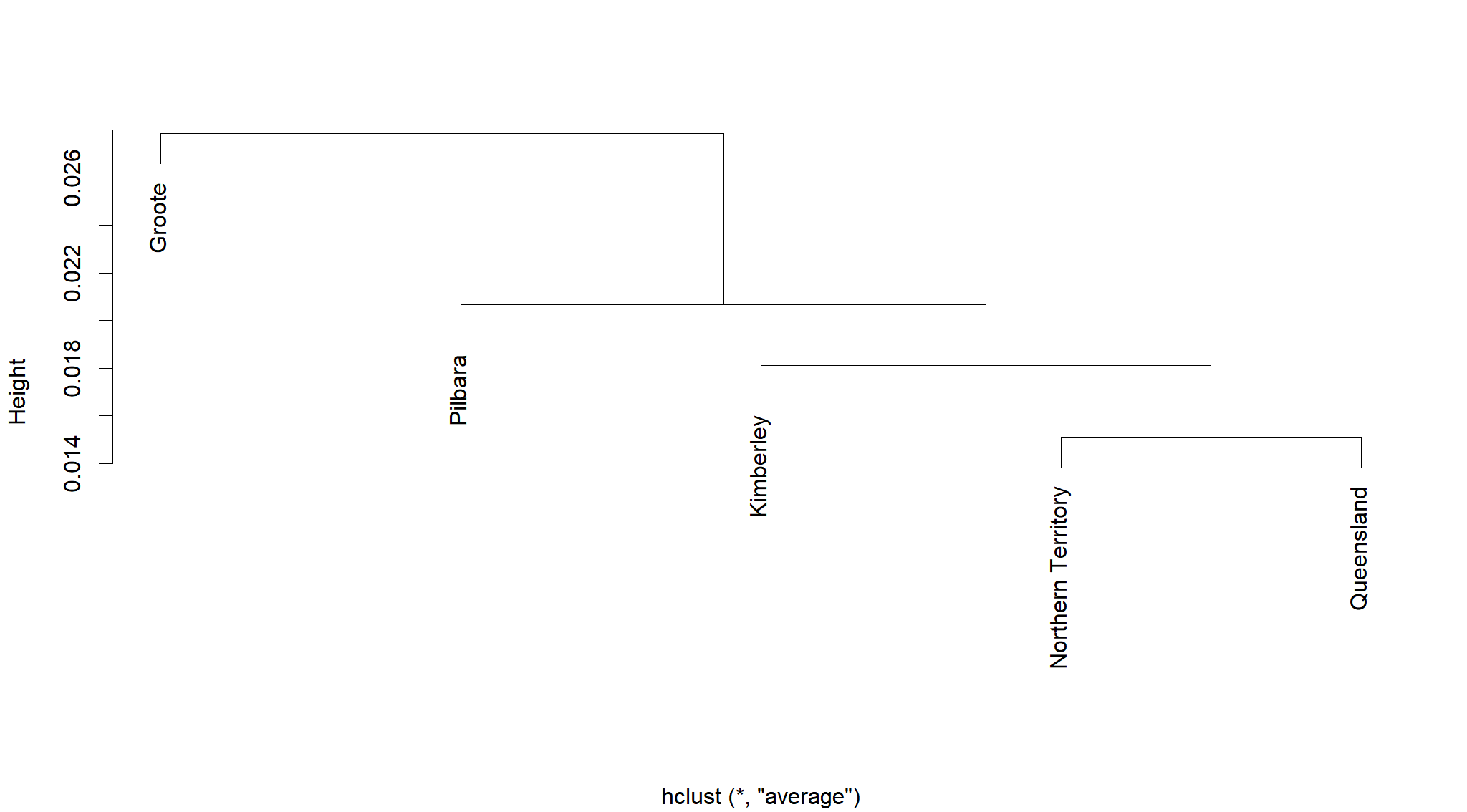


Figure S5: UPGMA clustering analysis of northern quoll populations based on Euclidean distances among mean shapes.


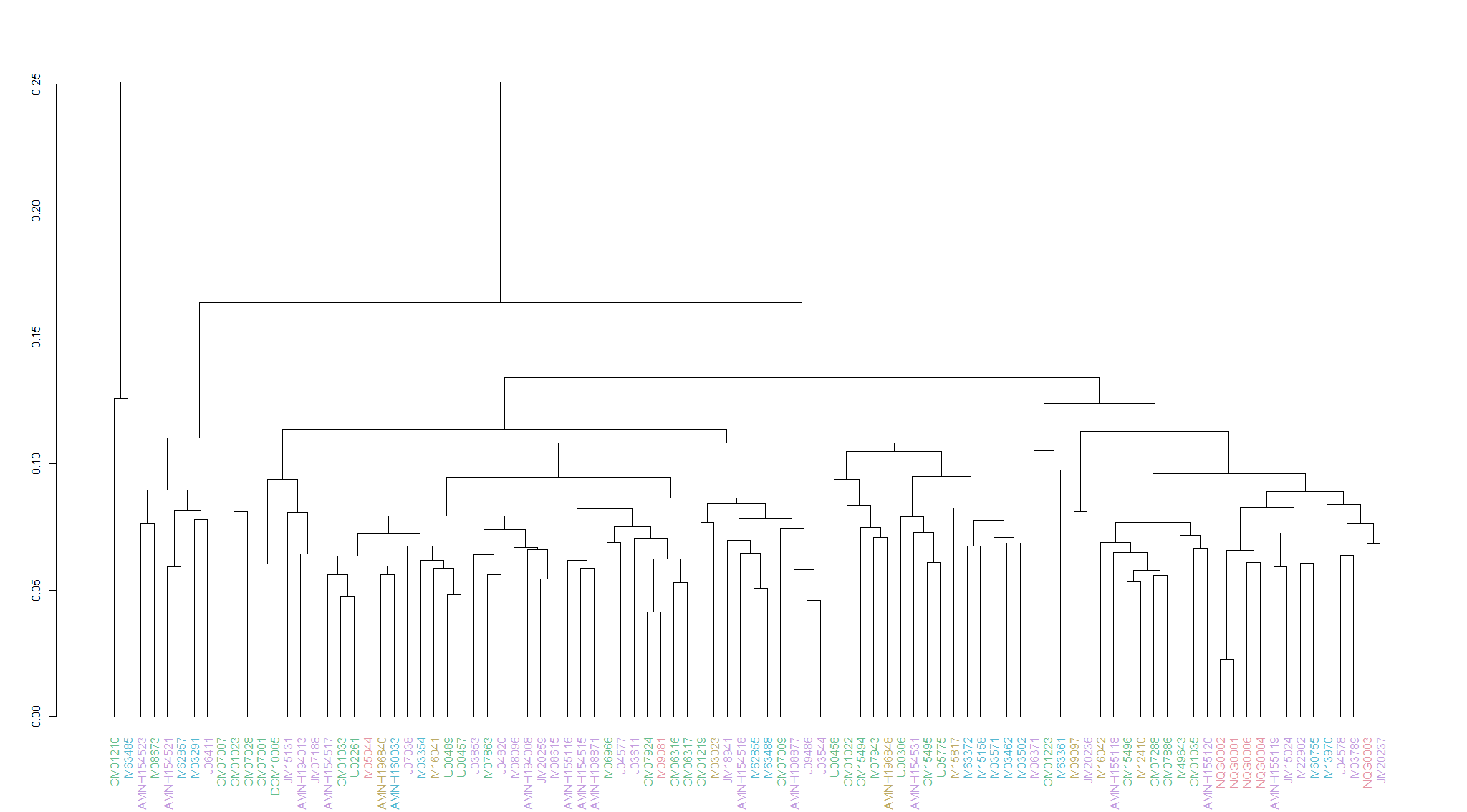


Figure S6: UPGMA clustering analysis of all northern quoll specimens based on Euclidean distances among specimens. Specimens are labelled by same colour pattern of populations as in Figure 2.
